# Efficacy and safety of EGFR-TKIs in combination with angiogenesis inhibitors as first-line therapy for advanced EGFR-mutant non-small-cell lung cancer: a systematic review and meta-analysis

**DOI:** 10.1186/s12890-023-02472-x

**Published:** 2023-06-14

**Authors:** Di Hu, Yan-Yan Zhou, Hong-Bo Ma, Miao-Miao Tao, Qun-Zhen Huang, Zhen-Zhou Yang, Qi Zhou

**Affiliations:** 1 The Second Affiliated Hospital of Chongqing Medical University, Chongqing, China; 2 Chongqing University Fuling Hospital, Chongqing, China; 3 Dalian Medical University, Liaoning, China

**Keywords:** NSCLC, EGFR mutation, EGFR-TKIs, Angiogenesis inhibitors, Meta-analysis

## Abstract

**Background:**

For patients with advanced non-small-cell lung cancer (NSCLC) with EGFR mutations, the suggested course of action is epidermal growth factor receptor-tyrosine kinase inhibitors (EGFR-TKIs). Even with a high disease control rate, a majority of patients develop acquired EGFR-TKIs resistance and eventually advance. To increase the benefits of treatment, clinical trials are increasingly exploring the value of EGFR-TKIs combined with angiogenesis inhibitors as a first-line treatment in advanced NSCLC carrying EGFR mutations.

**Method:**

Using PubMed, EMBASE and Cochrane Library, to locate published full-text articles in print or online, a thorough literature search was done from the database's inception to February 2021. Additionally, oral presentation RCTs from ESMO and ASCO were obtained. We sifted out RCTs that used EGFR-TKIs along with angiogenesis inhibitors as first-line therapy for advanced EGFR-mutant NSCLC. ORR, AEs, OS, and PFS were the endpoints. Review Manager version 5.4.1 was used for data analysis.

**Results:**

One thousand eight hundred twenty-one patients were involved in 9 RCTs. According to the results, combining EGFR-TKIs with angiogenesis inhibitors therapy prolonged PFS of advanced EGFR-mutation NSCLC patients on the whole [HR:0.65 (95%CI: 0.59~0.73, *P<*0.00001)]. No significant statistical difference was identified between the combination group and single drug group in OS(*P=*0.20) and ORR (*P=*0.11). There are more adverse effects when EGFR-TKIs are used in combination with angiogenesis inhibitors than when used alone.

**Conclusion:**

The combination of EGFR-TKIs and angiogenesis inhibitors prolonged PFS in patients with EGFR-mutant advanced NSCLC, but the OS and ORR benefit was not significant, and the risk of adverse events was higher, more pronounced with hypertension and proteinuria; PFS in subgroups suggested that the combination was associated with better PFS in the smoking, liver metastasis, and no brain metastasis groups, and the included studies suggested that the smoking group , liver metastasis group, and brain metastasis group may have a potential OS benefit.

## Main Text

### Introduction

The most prevalent malignancy in the world is lung cancer, with approximately 2.2million new cases and 1.79million new deaths due to lung cancer every year [1]. In China, the majority of cancer-related mortality and morbidity is caused by lung cancer [2], about 50% of lung adenocarcinoma patients have EGFR mutations, which always occur on 18 to 21 exons, and the EGFR exon-19 deletion (19del) and EGFR exon 21 L858R mutation (21 L858R) are the two mutations that are most frequently found [3, 4].

In numerous clinical trials, EGFR-TKIs showed a significant therapeutic advantage over traditional platinum-based chemotherapy, EGFR-TKIs demonstrated a strong clinical benefit, with median PFS extended to 9~18months and well tolerated. EGFR-TKIs are a first-line therapy for patients with advanced NSCLC who have sensitive EGFR mutations due to their efficacy and the lack of severe side effects. EGFR-TKIs from the first and second generations with acquired multidrug resistance make the long-term benefit a quagmire [4], Third-generation EGFR-TKIs administered as first-line therapy result in better PFS (FLAURA) [5, 6], but it also has a higher incidence and complexity of drug resistant [7], which created a great challenge for subsequent treatment. Because of the tumor heterogeneity, different treatments have different sensitivities to various tumor cells, that’s why we need combination therapy to cover more cell subsets or overcome the acquired EGFR-TKIs resistance.

Neovascularization can provide oxygen and nutrition to encourage metastasis and growth of tumor cells. Vascular endothelial growth factor (VEGF), a key regulator of angiogenesis in lung cancer, induced by hypoxia can stimulate proangiogenic signaling in conjunction with vascular endothelial growth factor receptor (VEGFR). The EGFR pathway can be activated to cause VEGF production and VEGFR activity, to promote angiogenesis through upregulation of hypoxia-dependent HIF-α expression [8], while the EFGR-TKIs directly suppresses tumor growth via preventing the EGFR pathway, and block the VEGF to inhibit angiogenesis. Blockade of VEGF/VEGFR signaling can reduce or erase the primary or acquired resistance to EGFR-TKIs [9, 10]. A growing number of clinical trials have tried to confirm that EGFR-TKIs combined with angiogenesis inhibitors have superior anti-tumor action than the EGFR-TKIs monotherapy in advanced EGFR-mutant NSCLC, but these studies did not achieve completely consistent results. In order to compare the effects of EGFR-TKIs combining angiogenesis inhibitors against EGFR-TKIs alone, we therefore aimed to comprehensively the published RCTs data to form a meta-analysis and systematic review. To achieve this, we specifically examined the PFS, OS, ORR as well as the incidence of serious adverse events, we also performed a subgroup analysis for these factors.

## Methods

### Search strategy

Through July 2021, we conducted an online search of PubMed, Embase, Cochrane Library and CNKI for publications describing EGFR-TKIs used in combination with angiogenesis inhibitors as the first-line treatment for NSCLC with EGFR mutation online through July 2021, and we also search the abstract accepted by European Society for Medical Oncology (ASCO) and European Society for Medical Oncology (EMSO) through May 2021. Search keywords included “non-small-cell lung cancer”, “NSCLC”, “anti-angiogenic”, “targeted therapy”, “clinical trial” and also their matching subject words.

### Inclusion and exclusion criteria

The following were the inclusion requirements: (1) Patients of NSCLC who had a biopsy confirm it. (2) Studies assessed the efficacy of EGFR-TKIs combining angiogenic inhibitors and EGFR-TKIs only as first-line treatment. (3) Studies reported one primary endpoint include PFS or OS, and reported one or more secondary endpoint. (4) Literature has a set of clear data of OS, PFS, ORR and incidence of adverse events, the hazard ratio (HR) and its 95% confidence interval (95%CI) can be obtained by calculation or directly from article.

The following were the exclusion requirements: (1) The study consisted of a single arm study. (2) We can’t get the data of primary or secondary endpoints. (3) Literature didn’t provide enough data or get full text. (4) The types of literature include case reports, conference abstracts, literature review, animal experiments, retrospective review.

### Study selection and data extraction

Two authors individually extracted the data, which were then placed into the typical datasheet. From the dataset, the following variables were taken out: the name of first author, publication year, trial’s abbreviation, journal, affiliation, study phase, country, interventional, format (full-text or abstract) and randomised controlled trials (RCTs), HR (PFS, OS, ORR), randomization methods, the randomized number of patients, the clinical and demographic data (gender, age, tumor, EGFR-mutant type), 95%CI of toxicity (3/4 grade). Any disagreement in extracted data was settled by consultation between two authors, if agreement could not be reached, the third author would make the final decision. If additional information is required, we will contact the authors of selected studies for the information needed. We will record it as Not Report (NR) if we still can’t obtain the information (Table 1).

**Table 1:** Literature search and study characteristic

**Investigator**	**Trail**	**Clinical Trial**	**Phase**	**Country**	**Treatment**	**EGFR Mutation (19Del; L858R;other)**	**Participants (Male; Female)**	**Smoking History (Never Smoker; Smoker)**	**pleural effusion (yes; no)**	**Brain metastasis (yes; no**	**liver metastasis (yes; no)**	**Clinical Stage (IIIB; IV;recurrence)**	**Baseline ECOG Score (0; 1)**	**Out** **-comes**
Hongyun Zhao	CTONG1706(ACTIVE) [11]	NCT02824458	III	China	Apatinib+ gefitinib	^a^81;74;2	^a^ 66;91	115;42	73;84	51;106	29;128	5;152;0	^a^48;107	PFS\ORR\AEs
					gefitinib	83;73;0	62;94	121;35	59;97	41;115	11;145	8;148;0	50;105	
Takashi Seto	JO25567 [12, 13]	JapicCTI-111390	II	Japan	Erlotinib+ bevacizumab	^a^40;35;0	^a^30;45	42;33	NR	NR	NR	NA	^a^43;32	PFS\OS\ORR\AEs
					erlotinib	40;37;0	26;51	45;32	NR	NR	NR	NA	41;36	
Haruhiro Saito	NEJ026 [12]	UMIN000017069	III	Japan	Erlotinib+ bevacizumab	^a^56;56;0	^a^41;71	^a^65;47	45;67	36;76	NR	^a^8;82;22	64;48	PFS\OS\ORR\AEs
					erlotinib	55;57;0	39;73	64;48	46;66	36;76	NR	8;84;20	68;42	
Qing Zhou	ARTEMIS-CTONG1509 [13]	NCT02759614	III	China	Erlotinib+ bevacizumab	^a^82;75;0	^a^60;97	NR	22;135	44;113	NR	^a^4;141;12	25;132	PFS\OS\ORR\AEs
					erlotinib	79;75;0	58;96	NR	41;113	47;107	NR	6;133;15	17;137	
Thomas E Stinchcombe	Stinchcombe [14]	NCT01532089	II	USA	Erlotinib+ bevacizumab	^a^NR	^a^NA	NA	NR	NA	NR	NR	NR	OS\AEs
					erlotinib	NR	NA	NA	NR	NA	NR	NR	NR	
Makoto Nishio	East Asian sunset of RELAY [15, 16]	NCT02411448	III	Japan	Ramucirumab +erlotinib	^a^84;80;0	^a^59;107	105;41	NR	NR	15;151	NR	86;80	PFS\OS\ORR\AEs
					Placebo+ erlotinib	84;86;0	61;109	109;52	NR	NR	19;151	NR	91;79	
Ponce Aix S	Europe/United States sunset of RELAY [15, 17]	NCT02411448	III	EU/US	Ramucirumab +erlotinib	^a^39;19;0	^a^24;34	29;29	NR	NR	NR	NR	30;28	PFS\ORR\AEs
					Placebo +erlotinib	36;19;0	22;33	30;25	NR	NR	NR	NR	28;27	
Hirotsugu Kenmotsu	WJOG9717L study [18]	NA	II	Japan	Osimertinib +bevacizumab	^a^35;26;0	^a^24;37	23;38	NR	18;43	6;55	^a^1;48;12	32;29	PFS\OS\ORR\AEs
					osimertinib	36;25;0	23;38	31;30	NR	23;38	11;50	2;46;13	34;27	
Maria Carmela Piccirillo	BEVERLY trial [19]	NA	III	Italy	Bevacizumab + erlotinib	^a^44;34;2	28;52	46;34	NR	NR	NR	3;77;0	^a^NR	PFS\OS\AEs
					erlotinib	44;32;4	30;50	37;43	NR	NR	NR	5;75;0	NR	

*Abbreviations*: *AEs* Adverse events, *ECOG* Eastern Cooperative Oncology Group, *EGFR* Epidermal growth factor receptor, *19del* EGFR exon 19 deletion, *L858R* EGFR exon 21 p.Leu858Arg, *NA* Not available, *NR* Not reported, *ORR* Objective response rate, *OS* Overall survival, *PFS* Progression-free survival, *RCT* Randomized controlled trial, *TKI* Tyrosine kinase inhibitor (erlotinib, gefitinib, or afatinib)

^a^Stratification factors

### Quality assessment

**Fig. 1:**
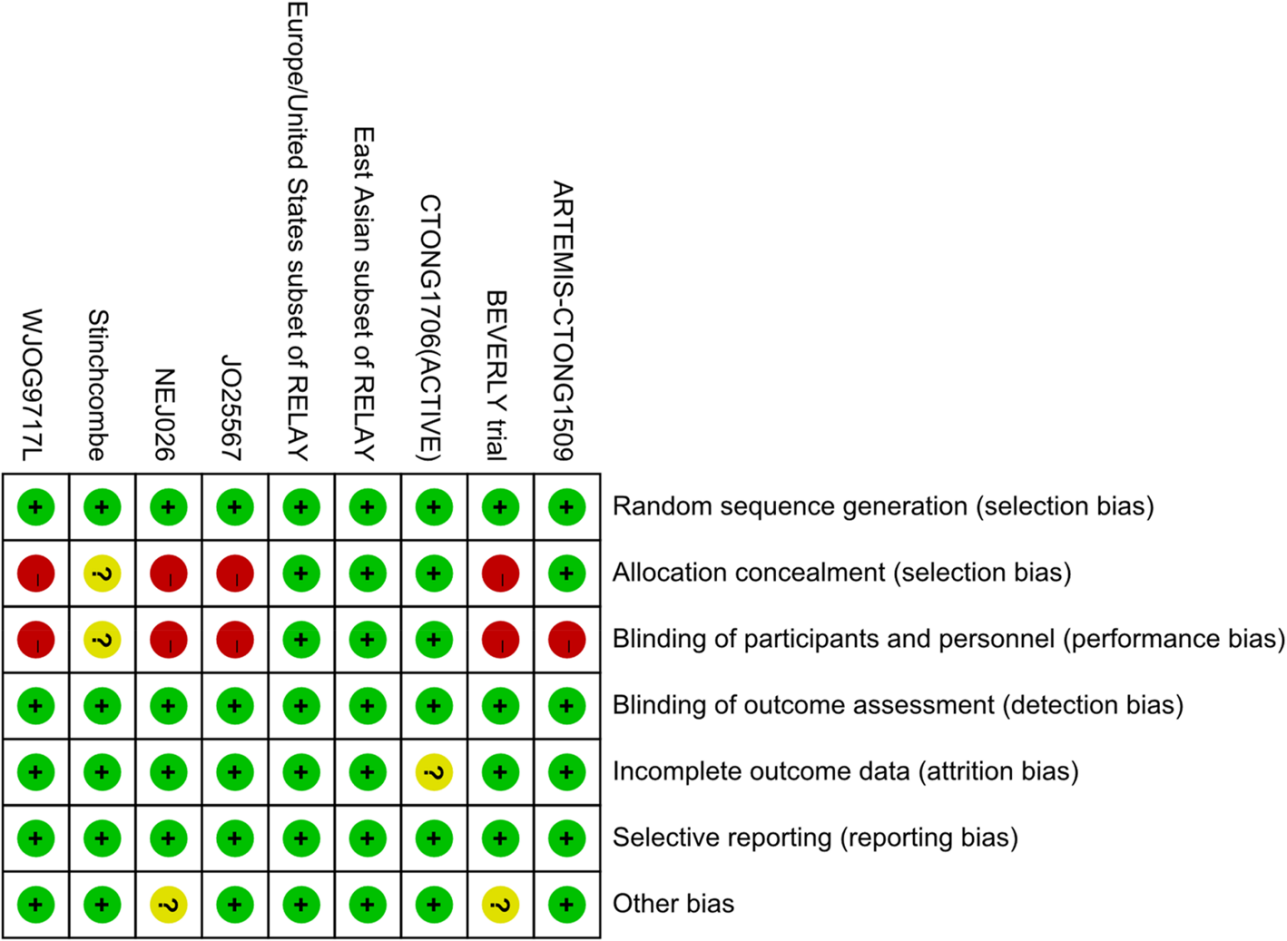
Risk of bias summary

Quality assessment was conducted for each of the eligible studies by using the Assessment of methodological quality tables (QUADAS), a risk-of-bios summary table (Fig. 1) was built in Review Manager (RevMan), version 5.4.1. According to the unified standard, the literatures are independently assessed by two investigators. They extracted and cross-checked these literatures, discussed and solved it in the case of disagreement.

### Statistical analysis

Data analysis was performed by Review Manager version 5.4.1. Count data chose the odds ratio (OR) or relative ratio (RR), and calculated the 95%CI. Bias among studies was assessed using the χ^2^ test, statistical significance criteria was *P*≤0.05. When *P*>0.05 or I^2^<50%, there is no significant difference among these studies, the fixed-effects model and random-effects model was used. To identify the sources of heterogeneity, a sensitivity analysis was conducted.

## Results

### Searching results

**Fig. 2:**
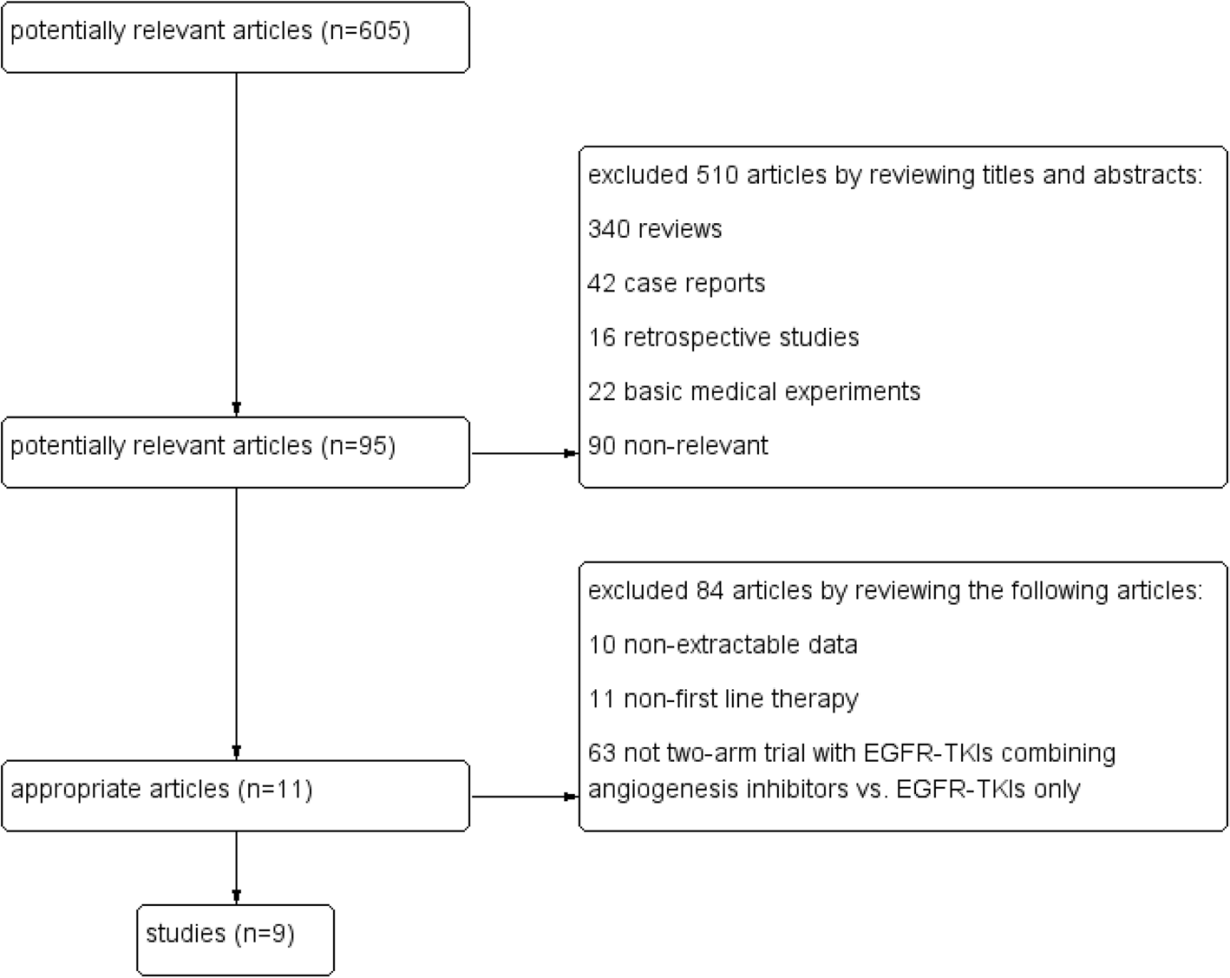
Flow chart of study selection

We searched these databases which we mentioned above and removed the duplicated to got 605 potentially relevant published articles. We got 95 articles after reviewing the titles and abstracts. We finally got 11 articles after intensively reading the full articles, and these articles included 9 studies with 1821 cases. The flow of literature screening is detailed in Fig. 2.

### Main characteristics of included articles

Nine studies included 1821 patients were enrolled for analyses. Table 1 lists the key characteristics of the studies that were included. Five of the included studies (JO25567 [15, 16], NEJ026 [12], ARTEMIS-CTONG1509 [13], Stinchcombe [14], BEVERLY trail [19]) Erlotinib combination with Bevacizumab as the first-line therapy was compared to Erlotinib alone in terms of effectiveness. The RELAY [17, 20, 21] study compared the efficiency of Ramucirumab combined Erlotinib with Erlotinib only in east Asian populations and European/US population separately, the CTONG1706 (ACTIVE) [11] study compared the Apatinib combine Gefitinib with Gefitinib only, the WJOG9717L [18] study compared the safety and efficacy of the Osimertinib combined Bevacizumab with Osimertinib only for advanced nonsquamous NSCLC. All studies described the tested EGFR mutant (exon-19del and exon-21 L858R mutation). Table 2 displays adverse events of grade 3/4.

**Table 2:** Severe adverse events

**Trail**	**Treatment**	**Rash**	**Diarrhoea**	**Proteinuria**	**Hypertension**	**Aminotransferase**
CTONG1706(ACTIVE)	Apatinib+ gefitinib	6/157	14/157	28/157	73/157	30/157
	gefitinib	1/154	2/154	1/154	4/154	21/154
JO25567	Erlotinib+ bevacizumab	19/75	1/75	6/75	45/75	6/75
	erlotinib	15/77	1/77	0/77	8/77	14/77
NEJ026	Erlotinib+ bevacizumab	23/112	6/112	8/112	26/112	9/112
	erlotinib	24/112	2/112	1/114	1/114	6/114
ARTEMIS-CTONG1509	Erlotinib+ bevacizumab	8/157	6/157	11/157	37/157	10/157
	erlotinib	6/153	0/153	0/153	10/153	12/153
Stinchcombe	Erlotinib+ bevacizumab	11/45	4/45	5/45	17/45	NR
	erlotinib	7/43	6/43	0/43	9/43	NR
East Asian sunset of RELAY	Ramucirumab +erlotinib	NR	9/164	4/164	35/164	22/164
	Placebo+ erlotinib	NR	2/170	0/170	8/170	24/170
Europe/United States sunset of RELAY	Ramucirumab +erlotinib	0/57	7/57	1/57	17/57	8/57
	Placebo +erlotinib	3/55	1/55	1/55	4/55	3/55
WJOG9717L study	Osimertinib +bevacizumab	0/61	0/61	2/61	4/61	0/61
	osimertinib	1/60	1/60	0/60	3/60	3/60
BEVERLY trial	Bevacizumab + erlotinib	25/80	NR	NR	19/80	NR
	erlotinib	11/80	NR	NR	4/80	NR

*Abbreviations*: *NR* Not reported

### Statistical pooling

#### Progression-free survival (PFS)

**Fig. 3:**
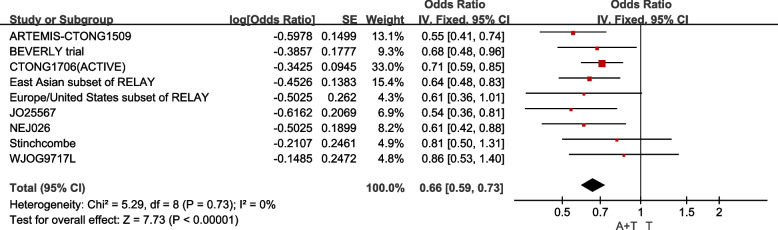
Median PFS in total population

Median PFS in total population is reported in 9studies, a total of 1821 individual patients enrolled, including combined therapy in 911patient and TKIs-only therapy in 910 patients. The fixed-effect model operated as I^2^= 0.0%, P for heterogeneity = 0.73. Compared with the EGFR-TKIs mono therapy, TKIs combined angiogenesis inhibitors therapy can prolonged the PFS of advanced EGFR-mutant NSCLC with statistical significance [HR:0.65 (95%CI: 0.59~0.73, *P<*0.00001)] (Fig. 3).

**Fig. 4:**
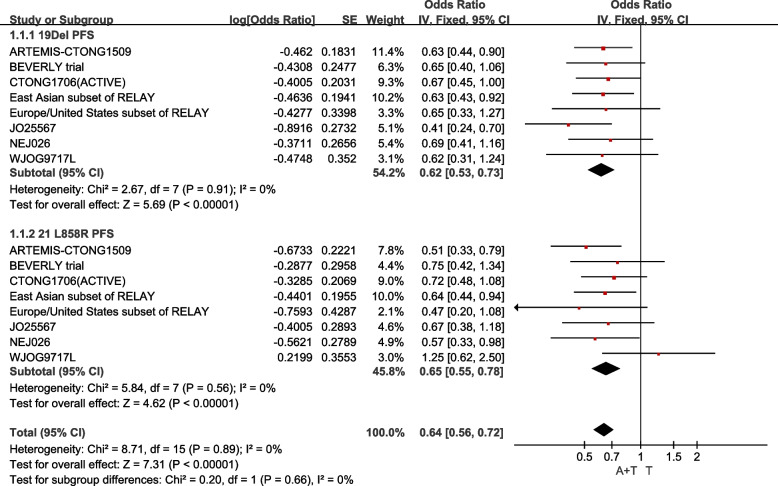
Median PFS of the population with 19Del mutation and 21L858R mutation

In the population with 19Del mutation, 918 cases included, the fixed-effect model operated as I^2^= 0.0%, P for heterogeneity = 0.91. It comes that the combining EGFR-TKIs with angiogenesis inhibitors therapy, compares to EGFR-TKIs monotherapy, prolonged the PFS of advanced NSCLC patients carrying 19Del mutation [HR:0.62 (95%CI: 0.53~0.73, *P<*0.00001)] (Fig. 3). And in the population with 21L858 mutation, included 803 cases, the fixed-effect model operated as I^2^= 0.0%, P for heterogeneity = 0.56. It comes that the therapy using EGFR-TKIs and angiogenesis inhibitors, contrasts with EGFR-TKIs only therapy, prolonged the PFS of advanced NSCLC patients carrying 21L858 mutation [HR:0.64 (95%CI: 0.56~0.72, *P<*0.00001)] (Fig. 4).

**Fig. 5:**
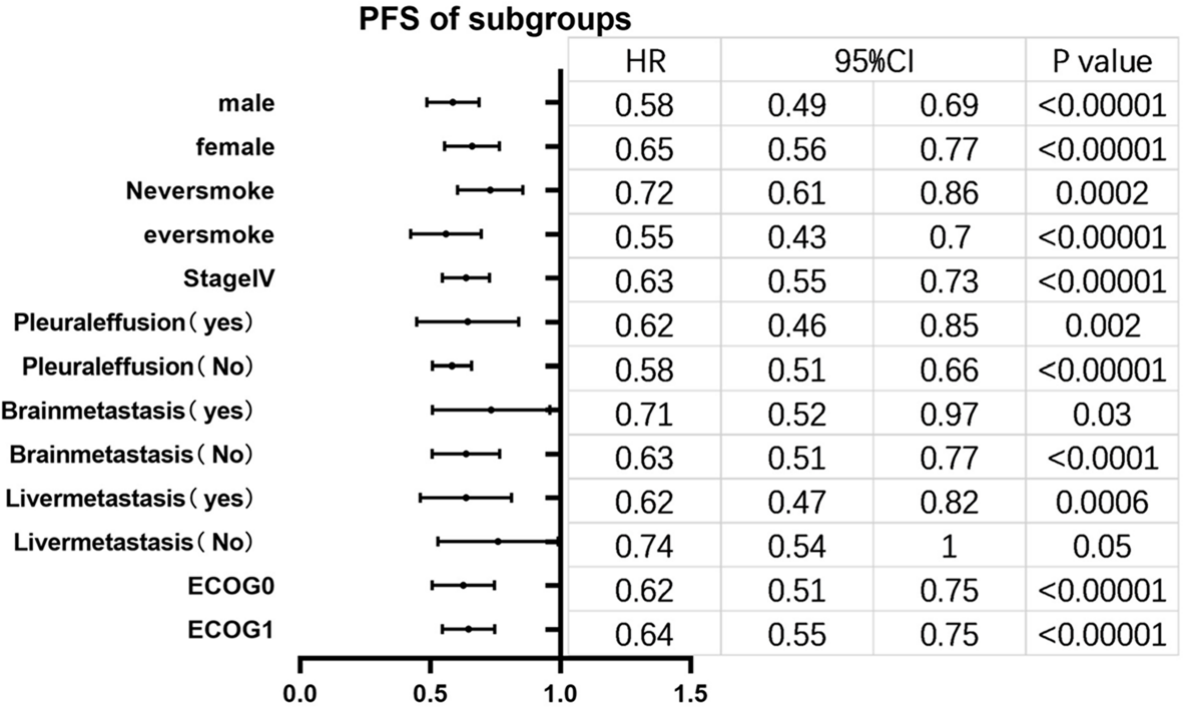
PFS of subgroups

Besides, those date were divided into different subgroups based on sexuality, stage at screening, baseline ECOG performance status, smoking status, brain metastasis, liver metastasis and pleural effusion. The results presented that the difference in non-baseline liver metastasis subgroup has no statistically significant (*P*>0.05), and the drug combination treatment has a longer PFS in other subgroups (Fig. 5). PFS prolongation was more obvious with the combination therapy in the male subgroup, ever smoke subgroup, non-baseline pleural effusion subgroup, non-baseline brain metastasis subgroup, baseline liver metastasis subgroup and the baseline ECOG 0 subgroup. The differences between the smoking group and the non-smoking group, the brain metastasis group and the no brain metastasis group, and the liver metastasis group and the no liver metastasis group were more obvious, which means that the prolongation of PFS was more obvious in the smoking group, the no brain metastasis group, and the liver metastasis group with the combination therapy.

#### Comparison of efficacy base on Overall survival (OS)

**Fig. 6:**
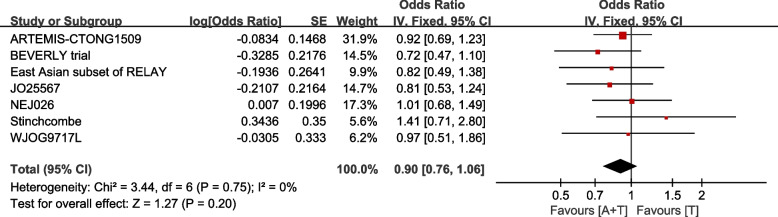
Overall survival (OS) in total population

OS is reported in 7 studies. The fixed-effect model operated as I^2^= 0.0%, P for heterogeneity = 0.75. No statistically significant difference was identify between the combination group and single drug group [HR:0.90(95%CI: 0.76-1.06, *P=*0.20)] (Fig. 6).

In the comparison between the ever smoke and never smoke subgroups, the combination therapy in the ever smoke subgroup was associated with better OS in the BEVERLY study (ever smoke subgroup: HR:0.41, 95% CI: 0.21~0.80; never smoke subgroup: HR:1.36, 95% CI: 0.70~2.64) and the difference is statistically significant (*P=*0.0077).

The OS results of two studies referred to baseline brain metastasis versus non-baseline brain metastasis subgroups, the baseline brain metastasis subgroup with the combination therapy in the ARTEMIS-CTONG 1509 study is associated with better OS, while no statistically significant difference was seen between the baseline brain metastasis and non-baseline brain metastasis subgroups in the Stinchcombe study.

#### Objective response rate (ORR)

**Fig. 7:**
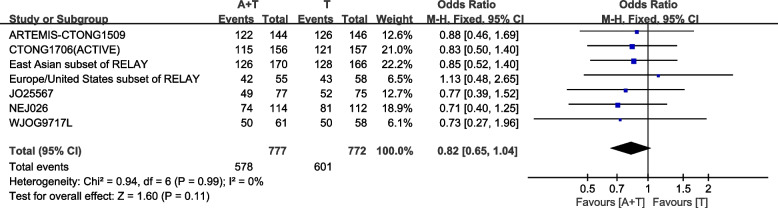
Objective response rate (ORR) in total population

ORR is reported in 7 studies. The fixed-effect model operated as I^2^= 0.0%, P for heterogeneity = 0.99. No statistically significant difference was identify between the combination group and single drug group[HR:1.21 (95%CI: 0.96-1.54, *P=*0.11)] (Fig. 7).

#### Severe adverse profile

**Fig. 8:**
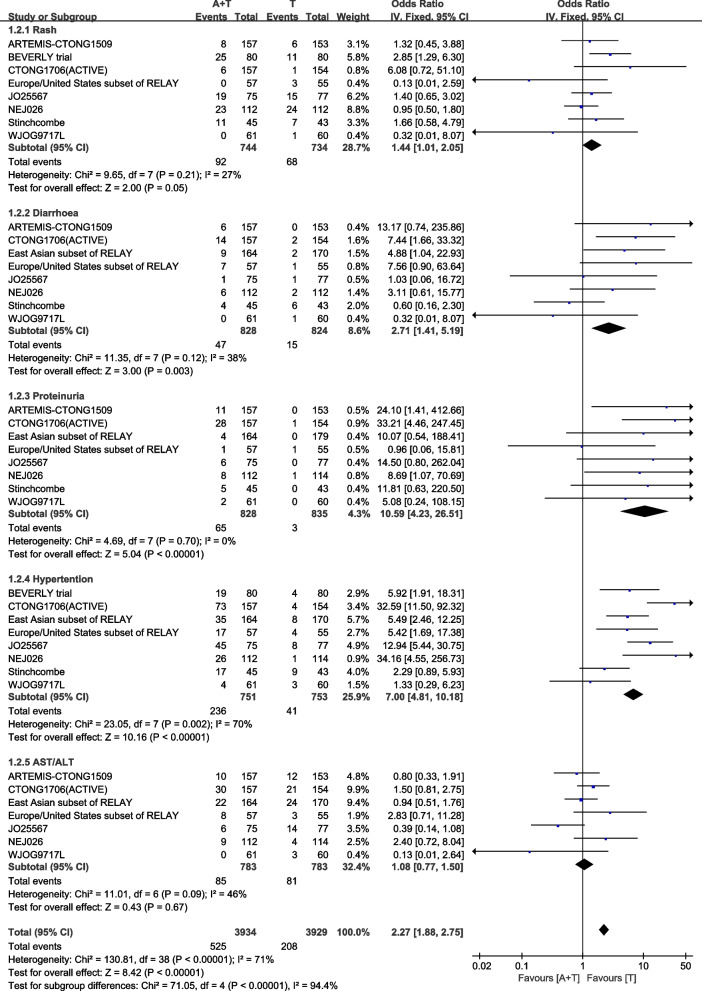
Adverse events (Grades≥3) in total population

Adverse Events (AEs) are reported in all 9 studies, the most common five AEs (Grades≥3) are rash with 160 patients, diarrhea with 62 patients, proteinuria with 68 patients, hypertension with 341 patients and abnormal ALT/AST with 166 patients. Except for the no significant statistical difference in abnormal ALT/AST group on both treatments (*P=*0.67), the incidences of rash[HR:1.44 (95%CI:1.01-2.05, *P=*0.05)], diarrhea[HR:2.71 (95%CI:1.41-5.19, *P=*0.003)], proteinuria[HR:10.59(95%CI: 4.23-26.51, *P<*0.00001)] and hypertension[HR:1.08 (95%CI: 0.77-1.50, *P<*0.00001)] on using angiogenesis inhibitors in conjunction with EGFR-TKIs group are all higher than the EGFR-TKIs only group. The incidence of adverse events was significantly higher in the proteinuria and hypertension subgroups compared to the other subgroups with combination therapy. The overall rate of incidence of adverse events for combining angiogenesis inhibitors with EGFR-TKIs treatment is still higher than the EGFR-TKIs only treatment[HR:2.43 (95%CI: 2.02-2.92, *P<*0.00001)] (Fig. 8).

### Publication bias and sensitivity analysis

Due to the limited numbers of included studies (*n<*10), we didn’t perform the publication bias analysis with Egger’s test. Using the sensitivity analysis for the high heterogeneity, the sensitivity analysis was conducted by sequentially removing trials, the results of outcome index still suggested a reliable.

## Discussion

Study limitations: (1) The number of RCTs that could be included in this study is limited, it may have led to bias in the results; (2) The RCTs we included have 6 studies with Asian including China and Japan, 2 studies with Europe (RELAY and BEVERLY trail) and 2 studies with US (RELAY and Stinchcombe); (3) Due to the limited number of included studies, the publication bias analysis didn’t perform in our study; (4) Due to the different stratification factors of each study, the results of our subgroup analysis are exploratory.

It has been demonstrated that the first-generation EGFR-TKIs have superior impact on extending OS and PFS compared with chemotherapy of EGFR-mutant NSCLC. Yet most patients experience disease progression, known as acquired resistance, around 11 months of EGFR-TKI therapy [22–24]. The vascular endothelial cells of the tumor stroma and the tumor cells themselves are simultaneously targeted and inhibited, which has a therapeutic synergistic effect. As a result, numerous randomized controlled trails comparing the effectiveness of EGFR-TKIs in combination with anti-angiogenic medicines to EGFR-TKIs alone in the first-line therapy of advanced NSCLC with the EGFR mutation have been successfully completed globally. The effectiveness and safety of EGFR-TKIs in combination with antiangiogenic medications in the first-line therapy of EGFR-mutant advanced NSCLC was therefore examined in a meta-analysis. Our results showed that EGFR-TKIs with angiogenesis inhibitors in combination significantly improved PFS, but had no effect on OS and ORR compared with EGFR-TKI plus placebo. Falling into the vicious circle of initial findings of targeted drugs that only prolong patient PFS, not OS.

Tumor vascular abnormalities and heterogeneity reduce drug delivery and reduce therapeutic efficacy. Preclinical studies [25, 26] have shown that acquired EGFR-TKI resistance is significantly dose-related. Dose was negatively correlated with the incidence of EGFR-TKI resistance. Additionally, it has been noted that angiogenesis inhibitors normalize tumour vasculature, anticancer drug absorption, enhancing tumor perfusion, and effectiveness of chemotherapy fortumor [9, 27]. However, the existing studies are the initial combination of EGFR-TKI and angiogenesis inhibitor for the treatment of EGFR-mutant advanced NSCLC. The rapid shrinkage of EGFR-TKI itself can lead to the reduction of the overall tumor blood vessels, masking the benefits of anti-angiogenesis. In clinical practice, when EGFR-TKI is used to treat NSCLC, the initial tumor shrinks rapidly, indicating that the drug concentration of EGFR-TKI is sufficient, and then there is a long-term SD persistent state. The drug concentration of TKI may achieve the purpose of prolonging OS.

Among patients with T790M mutation after EGFR-TKI application, the BOOSTER study rechallenged with Osimertinib and Bevacizumab included 155 patients and got a result of 55% objective response rate (ORR) and 90% disease control rate (DCR) with a median PFS of 15.4 months and median OS of 24 months [28]; Another phase I study included 25 patients rechallenged with Osimertinib and Ramucirumab and got a result of 87% ORR, 87% DCR in non-baseline CNS metastasis and 100% DCR in baseline CNS metastasis with a median PFS of 11.0 months and median OS of 25 months [29]. It shows that the different angiogenesis inhibitors have different effects on combination therapy, but we still need Further clinical trials to confirm it because of the differences in sample sizes, doses, or implementation processes across study procedures. Also the effect of combination therapy as re-challenge still has some potential benefit compared to EGFR-TKI alone. These studies suggest the need to explore the time window of using antiangiogenic in conjunction with EGFR-TKIs therapy.

The results of the subgroup analysis showed that in advanced EGFR-mutant NSCLC patients who had smoked previously, in advanced EGFR-mutant NSCLC, the addition of an angiogenesis inhibitor to EGFR TKI therapy resulted in statistically significant PFS and OS benefits that were comparable to those of EGFR-TKI alone, which is in contrast to the results of EGFR-TKI alone in the treatment of advanced NSCLC [30–32]. Tobacco exposure generates a heavy genomic mutational burden in lung cancer, including TP53 mutations and loss of liver kinase B1 (LKB1) expression [33–35]. Wild-type TP53 indirectly represses VEGF promoter activity by repressing transcription factors, such as SP1 and E2F, and there is also a TP53 binding site located within the VEGF promoter near the HIF-1α binding site, which is essential for VEGF induction during hypoxia. the association between TP53 mutation and increased VEGF-A transcripts is specific to lung adenocarcinoma. VEGF or VEGF receptor inhibitors have been linked to a better prognosis for tumors with TP53 mutations [36–38]. Subgroup analyses of RELAY randomized study comparing Erlotinib plus Ramucirumab to Erlotinib alone revealed that individuals with TP53 mutations had better survival rates [39]. The presence of TP53 mutations negatively affects the efficacy of single EGFR-TKI therapy. Patients with TP53 mutations had poorer PFS compared with those with wild-type TP53, the effectiveness of antiangiogenic and TKI therapy was unaffected, nevertheless. In patients with TP53 mutations, antiangiogenic coupled with TKI treatment was anticipated to considerably extend PFS compared to TKI alone (median PFS 15.0 vs. 8.0 months, *p <* 0.001), while no difference was observed in TP53 wild-type patients. These observations are also consistent with OS. Anti-angiogenic combined with TKI treatment resulted in significantly longer PFS and OS in patients with TP53 mutations detected in exons 5-8 compared to single TKI treatment [40]. Supporting the theory that antiangiogenic therapy is more effective in carrying TP53 mutations is the finding that EGFR-mutant NSCLC.

Tumor glycolysis is enhanced and attenuated by AMP-activated protein kinase (AMPK)-dependent inhibition of mTOR in NSCLC patients who smoke; this in turn inhibits expression of the master kinase of the AMPK subfamily, LKB1, through CpG island methylation, and LKB1 expression was positively correlated with the sensitivity of NSCLC patients to TKIs [41]. Loss of LKB1 causes intricate alterations in the microenvironment, supporting a role in the control of angiogenesis and pointing to a potential role in the response to anti-angiogenic therapy [42, 43]. These findings suggest that EGFR-TKI combined with antiangiogenic therapy may still have a survival benefit in some types of EGFR-mutant NSLC patients, requiring further stratification. Prospective clinical trials must also be used to confirm it.

The current investigation shows that metastases in pleural, liver and bones are independent risk factors for death. However, in patients who received antiangiogenics during treatment, there was no discernible difference in median OS between groups with and without pleural, liver, and bone metastases. Liver metastasis as independent poor predictive factors of outcome [43], the efficacy of EGFR-TKIs decreases in NSCLC patients with liver metastasis [44]. Our study shows that combination therapy has a large efficacy improvement in individuals with liver metastasis than in patients without liver metastasis (baseline liver metastasis: HR:0.62, 95%CI:0.47~0.82, *P=*0.0006; non-baseline liver metastasis: HR:0.74, 95%CI: 0.54~1.00, *P=*0.05). The immune suppressive microenvironments of liver are included the regulatory T cells (T regs) and myeloid-derived suppressor cells (MDSC), these cells are crucial in the liver’s promotion of metastatic spread. Treatment with Bevacizumab normalizes vasculature [45] to reduce the T regs and depress the activities of T regs and MDSCs [46]. The angiogenesis inhibitors can improve the sensitivity of EGFR-TKIs [47] and show the synergistic effect [48], these could be the reason that the combination therapy has a better efficacy improvement in patients with liver metastasis. Brain metastasis is a poor predictor of outcome for EGFR-TKI monotherapy in NSCLC. However, our results showed that EGFR-TKI plus angiogenesis inhibitor therapy in NSCLC with non-baseline brain metastases prolonged PFS significantly compared with EGFR-TKI monotherapy (baseline brain metastasis: HR: 0.71, 95%CI: 0.52~0.97, *P=*0.03; non-baseline brain metastasis: HR: 0.63, 95%CI: 0.51~0.77, *P<*0.0001). However, the two included studies that mentioned OS outcomes in the brain metastasis subgroups suggested a possible OS benefit. Additionally, Tao Jiang and colleagues reported that EGFR-TKI plus Bevacizumab not only had longer intracranial PFS (14.0 vs. 8.2 months) and systemic PFS (14.4 vs. 9.0 months), but also prolonged OS by the inclusion of Bevacizumab (29.6 months vs. 21.7 months; *P* < 0.001). Moreover, it improved intracranial versus systemic ORR. An independent relationship between the addition of Bevacizumab and prolonged intracranial and systemic PFS and OS was found by multivariate analysis [49].

In the statistics of the incidence of adverse events (Grade ≥3), the incidence of hypertension and proteinuria was significantly higher in the combination therapy. The incidence of hypertension, the most common adverse event of the cardiovascular system with angiogenesis inhibitors, unsurprisingly showed a large difference between the two groups. Patients with a history of hypertension during angiogenesis inhibitor therapy are more likely to develop severe hypertension [50], and in several RCTs included in our study, patients' baseline blood pressure was not statistically detailed, and it cannot be excluded that patients with baseline hypertensive disease increased the severity of hypertension further after treatment. It has also been shown that plasma VEGF-A concentrations are associated with the development of hypertension after angiogenesis inhibitor used, with increased plasma levels of VEGF-A observed in treatment with VEGF pathway inhibitor and with insufficient NO production by endothelial cells to cause adequate vasodilation in a subgroup of patients with low VEGF-A levels, and that treatment with bevacizumab in these patients may further limit NO release from endothelial cells and other vasodilators, leading to severe hypertension after treatment. In contrast, patients with high VEGF-A levels have relative protection from severe hypertension after treatment with bevacizumab [51]. The severity of the occurrence of hypertensive adverse events in relation to the presence of baseline hypertensive disease and baseline plasma VEGF-A levels needs to be confirmed by further stratification studies.

The incidence of adverse events in proteinuria is similarly correlated with the dose of anti-angiogenic drugs. Binding of VEGF produced by renal podocytes to VEGFR on glomerular endothelial cells is essential for the induction and maintenance of endothelial cell window holes and selective depletion of VEGF in podocytes during the use of angiogenesis inhibitors leads to proteinuria. The loss of the protective effect of VEGF also activates the endothelin-1 (ET-1) pathway allowing the loss of renin from the podocytes and contributing to the development of proteinuria [50]. The east Asian subset and the Europe/United States subset of RELAY study which we included showed a significant difference. Our results suggest that the incidence of proteinuria with Grade ≥3 is significantly higher in east Asian populations compared to European and American populations. Given that EGFR mutations are common in Asian populations, whether this adverse effect of proteinuria is ethnically related needs further investigation.

## Conclusion

Compared with the EGFR-TKIs only therapy, the therapy of angiogenesis inhibitors with EGFR-TKIs together prolonged the PFS of advanced EGFR-mutation NSCLC patients. Even the combination therapy showed no obvious benefit in OS and ORR, the high risk of incidence of adverse events in combined therapy, more obvious with hypertension and proteinuria. But the PFS of subgroups suggested that the combination therapy is associated with better PFS in the ever smoke, baseline liver metastasis, and non-baseline brain metastasis subgroups, and the included studies suggested the potential OS benefits in ever smoke, baseline liver metastasis and baseline brain metastasis subgroups. It needs to be consideration of baseline brain metastasis, baseline liver metastasis, smoking, baseline hypertensive, renal function, and ethnicity into the stratification factors, and build a large prospective study to validate the findings, which will help the development of clinical therapy strategies .

## Data Availability

The datasets generated during or analysed during the current study are included in the article.

## Abbreviations

19del: EGFR exon 19 deletion
21 L858R: EGFR exon 21 L858R mutation
AEs: Adverse events
AMPK: AMP-activated protein kinase
ASCO: American Society of Clinical Oncology
che + bev: chemotherapy plus bevacizumab
DCR: Disease Control Rate
ECOG: Eastern Cooperative Oncology Group
EGFR: Epidermal growth factor receptor
EGFR-TKIs: Epidermal growth factor receptor-tyrosine kinase inhibitors
ESMO: European Society for Medical Oncology
HR: Hazard ratio
LKB1: Liver kinase B1
MDSCs: Myeloid-derived suppressor monocytes
NA: Not available
NR: Not reported
NSCLC: Non-small-cell lung cancer
OR: Odds ratio
ORR: Objective response rate
osi+ bev: Osimertinib plus bevacizumab
OS: overall survival
PFS: Progression-free survival
RCT: Randomized controlled trial
RevMan: Review Manager
RR: Relative ratio
TKI: Tyrosine kinase inhibitor
T regs: Regulatory T cells
